# Supplementary material for: MetaQTL: a package of new computational methods for the meta-analysis of QTL mapping experiments
Source: BMC Bioinformatics. 2007 Feb 8;8:49. doi: 10.1186/1471-2105-8-49 (PMC1808479; doi:10.1186/1471-2105-8-49)
Supplement: Additional File 7 — MetaQTL Package : jar file and tutorial. This Zip archive contains both the MetaQTL JAR file and the files of the tutorial. [file 1471-2105-8-49-S7.zip › org.inra.metaqtl/doc/org/metaqtl/main/package-tree.html]

org.metaqtl.main Class Hierarchy


|  |  |  |
| --- | --- | --- |
| **Overview** \| **Package** \| Class \| Use \| **Tree** \| **Deprecated** \| **Index** \| **Help** | |  |
| **PREV**   **NEXT** | **FRAMES**    **NO FRAMES**     **All Classes** |  |


---


## Hierarchy For Package org.metaqtl.main

**Package Hierarchies:**: All Packages

---

## Class Hierarchy

- java.lang.Object
  - org.metaqtl.main.**CmdLineParser**- org.metaqtl.main.**CmdLineParser.Option**
      - org.metaqtl.main.**CmdLineParser.Option.BooleanOption**- org.metaqtl.main.**CmdLineParser.Option.DoubleOption**- org.metaqtl.main.**CmdLineParser.Option.IntegerOption**- org.metaqtl.main.**CmdLineParser.Option.LongOption**- org.metaqtl.main.**CmdLineParser.Option.StringOption**
      - org.metaqtl.main.**MetaMain**
        - org.metaqtl.main.**A2Xml**- org.metaqtl.main.**ConsMap**- org.metaqtl.main.**InfoMap**- org.metaqtl.main.**MapView**- org.metaqtl.main.**MetaDB**- org.metaqtl.main.**MMapView**- org.metaqtl.main.**MQTLView**- org.metaqtl.main.**QTLClust**- org.metaqtl.main.**QTLClustInfo**- org.metaqtl.main.**QTLModel**- org.metaqtl.main.**QTLProj**- org.metaqtl.main.**QTLTree**- org.metaqtl.main.**Xml2A**
        - java.lang.Throwable (implements java.io.Serializable)
          - java.lang.Exception
            - org.metaqtl.main.**CmdLineParser.OptionException**
              - org.metaqtl.main.**CmdLineParser.IllegalOptionValueException**- org.metaqtl.main.**CmdLineParser.UnknownOptionException**
                  - org.metaqtl.main.**CmdLineParser.NotFlagException**- org.metaqtl.main.**CmdLineParser.UnknownSuboptionException**

---


|  |  |  |
| --- | --- | --- |
| **Overview** \| **Package** \| Class \| Use \| **Tree** \| **Deprecated** \| **Index** \| **Help** | |  |
| **PREV**   **NEXT** | **FRAMES**    **NO FRAMES**     **All Classes** |  |


---
